# Factors associated with violent behavior among hospitalized adolescents with affective disorders: a two-center cross-sectional study

**DOI:** 10.3389/fpsyt.2026.1905434

**Published:** 2026-08-19

**Authors:** Yunge Zhang, Heying Cheng, Chunxiao Li, Hang Yu, Wei Jiang, Guangjian Li, Weixiong Cai

**Affiliations:** 1 Shanghai Key Laboratory of Forensic Medicine, Key Laboratory of Forensic Science, Ministry of Justice, Academy of Forensic Science, Shanghai, China; 2 Forensic Identification Institute, Changzhou Dean Hospital, Changzhou, Jiangsu, China; 3 Department of Psychiatry, Wuhu Fourth People’s Hospital, Wuhu, Anhui, China; 4 Graduate School, Wannan Medical University, Wuhu, Anhui, China; 5 Department of Psychology, Changzhou Dean Hospital, Changzhou, Jiangsu, China

**Keywords:** adolescents, affective disorders, associated factors, psychiatric inpatients, violent behavior

## Abstract

**Objective:**

Violent behavior among hospitalized adolescents with affective disorders poses substantial challenges to psychiatric care and ward safety. This study aimed to examine demographic, clinical, behavioral, family-related, and personality-related factors associated with violent behavior among hospitalized adolescents with affective disorders.

**Methods:**

A two-center cross-sectional study was conducted among 202 adolescents with affective disorders admitted to two psychiatric hospitals in China. Violent behavior was assessed using the Modified Overt Aggression Scale (MOAS), with a total score of ≥4 indicating the presence of violent behavior. Non-suicidal self-injury (NSSI), anxiety and depressive symptoms, and personality traits were assessed using standardized instruments. Variables for the multivariable logistic regression analysis were selected based on previous evidence, clinical relevance, the conceptual framework of the study, and the available number of violent events. Study center, age, sex, diagnostic category, single-parent family status, NSSI score, GAD-7 score, neuroticism, and conscientiousness were entered simultaneously into the adjusted model.

**Results:**

Among the 202 participants, 74 (36.6%) met the criterion for violent behavior. After adjustment for the other variables in the model, single-parent family status was associated with higher odds of violent behavior (OR = 3.709, 95% CI: 1.535–8.965), as was bipolar disorder compared with major depressive disorder (OR = 2.879, 95% CI: 1.030–8.046). Male participants had higher odds of violent behavior than female participants (OR = 3.009, 95% CI: 1.321–6.855). Higher NSSI scores (OR = 1.073, 95% CI: 1.033–1.116) and higher neuroticism scores (OR = 1.322, 95% CI: 1.047–1.670) were associated with higher odds of violent behavior, whereas higher conscientiousness scores were associated with lower odds of violent behavior (OR = 0.695, 95% CI: 0.552–0.875).

**Conclusions:**

Violent behavior was common in this sample of hospitalized adolescents with affective disorders. Sex, family structure, diagnostic category, NSSI, neuroticism, and conscientiousness were independently associated with concurrent violent behavior. These findings provide a multidimensional understanding of the characteristics associated with violent behavior in adolescent psychiatric inpatient settings. Because of the cross-sectional design, the findings should not be interpreted as evidence of causal relationships or prospective predictive validity.

## 1 Introduction

Affective disorders are common psychiatric conditions characterized primarily by disturbances in mood, including major depressive disorder and bipolar disorder. Their clinical manifestations involve impairments across emotional, cognitive, and behavioral domains (1, 2). These disorders typically emerge during adolescence and early adulthood, and their prevalence among adolescents has increased in recent years, making them a major public health concern affecting youth mental health (3–5). Previous studies have shown that individuals with affective disorders often experience difficulties in emotion regulation, impaired impulse control, and reduced social functioning (6, 7). These characteristics have been associated with aggressive and violent behaviors (8). Violent behavior can adversely affect treatment adherence and clinical outcomes and may also compromise the safety of healthcare staff, disrupt family relationships, and contribute to broader social burdens (9). Therefore, clarifying the factors associated with violent behavior among hospitalized adolescents with affective disorders is of considerable clinical importance (10).

Previous research has shown that violent behavior is associated with a combination of individual and environmental factors, including emotional symptoms, childhood adversity, personality traits, and family-related factors (11, 12). Other established correlates of violence include previous violent or criminal behavior and substance misuse, while childhood maltreatment and exposure to domestic violence have been associated with antisocial and violent behavior during adolescence (13, 14). At the individual level, emotional disturbances, personality characteristics, and behavioral problems have all been associated with aggression and violence (15, 16). At the environmental level, parenting practices, parent–child relationships, and the broader family environment may also be associated with adolescent behavioral patterns (17). However, existing findings have been inconsistent across populations, and the interplay among these factors remains incompletely understood. Given that violent behavior is associated with multiple individual, clinical, behavioral, and environmental characteristics, examining individual factors in isolation may not adequately capture its complexity. Therefore, the simultaneous examination of factors across multiple domains is warranted.

Although previous studies have provided valuable insights into the characteristics associated with violent behavior, several gaps remain. First, most investigations have focused on community-based adolescents or adults with psychiatric disorders, whereas evidence regarding hospitalized adolescents with affective disorders remains limited. Adolescent psychiatric inpatient settings frequently involve acute behavioral dysregulation and aggressive incidents that require clinical intervention, making the assessment and management of violent behavior particularly important in this population (18). As this population is often characterized by more severe emotional symptoms, greater impulsivity, and a higher burden of behavioral problems, the factors associated with violent behavior may differ from those observed in other groups. Second, previous studies have largely examined the relationship between individual characteristics and violent behavior, with relatively little attention given to the simultaneous examination of demographic, clinical, behavioral, personality-related, and family-related factors among hospitalized adolescents. Finally, although structured violence risk assessment approaches have been used in psychiatric inpatient settings (19), such tools are conceptually different from cross-sectional analyses of factors associated with concurrent violent behavior. Evidence specifically focusing on hospitalized adolescents with affective disorders remains limited, particularly in non-Western clinical settings.

Against this background, the present study focused on hospitalized adolescents with affective disorders. We examined factors associated with violent behavior from multiple domains, including demographic characteristics, clinical factors, behavioral characteristics, personality traits, and family structure. Given the cross-sectional design, the present study aimed to identify factors associated with concurrent violent behavior rather than to establish causal relationships or predict future violent events. The findings may provide additional evidence regarding the multidimensional characteristics associated with violent behavior in this specific psychiatric inpatient population and inform future longitudinal research.

## 2 Methods

### 2.1 Participants

This two-center cross-sectional study was conducted at Changzhou Dean Hospital and Wuhu Fourth People’s Hospital. A total of 202 adolescents with affective disorders who were hospitalized between June 2025 and April 2026 were consecutively recruited.

The study protocol was approved by the Ethics Committee of Changzhou Dean Hospital (Approval No. CZDALL-2025-001) and the Ethics Committee of Wuhu Fourth People’s Hospital (Approval No. 2025-KY-028). Written informed consent was obtained from all participants and their legal guardians prior to enrollment. All procedures were conducted in accordance with the principles of the Declaration of Helsinki.

Participants were eligible if they:(1) met the diagnostic criteria for major depressive disorder or bipolar disorder according to the International Classification of Diseases, 10th Revision (ICD-10); (2) were aged between 13 and 18 years; (3) were receiving inpatient psychiatric treatment and were able to complete the study assessments; (4) provided written informed consent, together with consent from their legal guardians. Participants were excluded if they: (1) had severe neurological disorders or serious physical illnesses; (2) had intellectual disability or severe cognitive impairment; (3) had other major psychiatric disorders, such as schizophrenia or substance use disorders; (4) had incomplete clinical data.

### 2.2 Instruments

#### 2.2.1 General information

Demographic and clinical information, including study center, sex, age, educational level, family history, first-admission status, only-child status, single-parent family status, and diagnostic category, was obtained from structured questionnaires and medical records.

#### 2.2.2 Assessment of violent behavior

Violent behavior was assessed using the Modified Overt Aggression Scale (MOAS). The MOAS was developed by Kay et al. based on the Overt Aggression Scale and consists of four dimensions: verbal aggression, aggression against property, autoaggression, and physical aggression. Each dimension is rated on a scale from 0 to 4 according to the severity of aggressive behavior. Different weights are assigned to each dimension, and the weighted total score is calculated as follows: MOAS total score = verbal aggression × 1 + aggression against property × 2 + autoaggression × 3 + physical aggression × 4. Higher scores indicate more severe aggressive behavior (20). The Chinese version of the MOAS has demonstrated good reliability and validity, with a Cronbach’s α coefficient of 0.842 (21). Based on previous research involving adolescents with depressive disorders, a total MOAS score of ≥4 was used as an operational cutoff to classify participants according to the presence or absence of violent behavior (22). Participants with MOAS scores ≥4 were classified as having violent behavior, whereas those with scores <4 were classified as not having violent behavior. Because prevalence estimates may vary according to the selected cutoff, the potential influence of this operational definition is acknowledged in the Discussion.

#### 2.2.3 Assessment of depressive symptoms

Depressive symptoms were assessed using the Patient Health Questionnaire-9 (PHQ-9). The PHQ-9 was developed by Kroenke et al. and consists of nine items. Each item is rated on a scale from 0 to 3, yielding a total score ranging from 0 to 27, with higher scores indicating greater severity of depressive symptoms (23). The Chinese version of the PHQ-9 has demonstrated good reliability and validity, with a Cronbach’s α coefficient of 0.860 (24).

#### 2.2.4 Assessment of anxiety symptoms

Anxiety symptoms were assessed using the Generalized Anxiety Disorder-7 (GAD-7). The GAD-7 was developed by Spitzer et al. and consists of seven items. Each item is rated on a 4-point scale (0–3), resulting in a total score ranging from 0 to 21, with higher scores indicating more severe anxiety symptoms (25). The Chinese version of the GAD-7 has demonstrated good reliability and validity, with a Cronbach’s α coefficient of 0.840 (26).

#### 2.2.5 Assessment of personality traits

Personality traits were assessed using the 10-item version of the Big Five Inventory (BFI-10). The BFI-10 was developed by Rammstedt et al. and consists of 10 items covering five personality dimensions: extraversion, agreeableness, conscientiousness, neuroticism, and openness, with each dimension represented by two items. All items are rated on a 5-point Likert scale, and higher scores indicate a stronger expression of the corresponding personality trait (27). The BFI-10 has been used in Chinese samples and has shown acceptable internal consistency, with a Cronbach’s α coefficient of 0.740 (28).

#### 2.2.6 Assessment of non-suicidal self-injury

Non-suicidal self-injury (NSSI) was assessed using the Adolescent Non-Suicidal Self-Injury Assessment Questionnaire (ANSAQ), developed by Wan Yuhui et al. The questionnaire was used to evaluate the occurrence of NSSI behaviors during the previous 12 months. It consists of 12 items covering common forms of self-injurious behavior, including self-pinching, self-scratching, hitting hard objects, self-biting, self-cutting, and self-burning or scalding. Each item is rated on a 5-point Likert scale (0 = never, 1 = occasionally, 2 = sometimes, 3 = often, and 4 = always). Higher total scores indicate greater severity of NSSI behaviors. The ANSAQ has demonstrated good reliability and validity, with a Cronbach’s α coefficient of 0.921 (29).

### 2.3 Statistical analysis

Statistical analyses were performed using SPSS version 26.0. Continuous variables were tested for normality before analysis. Normally distributed data are presented as mean ± standard deviation and were compared between groups using the independent-samples t-test. Categorical variables are presented as frequencies and percentages [n (%)] and were compared using the chi-square test or Fisher’s exact test, as appropriate.

Violent behavior was treated as the dependent variable (0 = no, 1 = yes). Variables for the primary multivariable logistic regression model were selected according to previous evidence, clinical relevance, the conceptual framework of the study, and the available number of violent events, rather than solely according to statistical significance in the univariate analyses. Study center, age, and sex were included as adjustment variables to account for potential center-related and demographic differences. Diagnostic category, single-parent family status, NSSI score, GAD-7 score, neuroticism, and conscientiousness were included because they represented the principal clinical, family-related, behavioral, emotional, and personality-related domains examined in this study. All nine variables were entered simultaneously using the enter method and retained in the model irrespective of their individual P values. Given that only 74 participants met the criterion for violent behavior, additional measured variables were not entered into the primary model to reduce the risk of overfitting and unstable estimates.

Multicollinearity among the independent variables was assessed using tolerance and variance inflation factor (VIF) values. Tolerance values greater than 0.20 and VIF values below 5 were considered indicative of no serious multicollinearity. Overall model fit was assessed using the omnibus likelihood-ratio test, the Hosmer–Lemeshow goodness-of-fit test, and Nagelkerke’s R². Odds ratios (ORs) and their corresponding 95% confidence intervals (95% CIs) were calculated. A two-sided P value <0.05 was considered statistically significant.

## 3 Results

### 3.1 Demographic characteristics and univariate analysis

A total of 202 hospitalized adolescents with affective disorders were included. Among them, 74 participants (36.6%) had MOAS scores ≥4 and were classified as having violent behavior, whereas 128 participants (63.4%) had MOAS scores <4 and were classified as not having violent behavior.

No statistically significant differences were observed between the two groups in age, sex, study center, family history, educational level, first-admission status, only-child status, depressive symptoms, extraversion, openness, or agreeableness (all P > 0.05). Significant between-group differences were observed in diagnostic category, single-parent family status, NSSI score, GAD-7 score, neuroticism, and conscientiousness (all P < 0.05).

Compared with participants without violent behavior, those with violent behavior were more frequently diagnosed with bipolar disorder and were more likely to come from single-parent families. They also had higher NSSI, GAD-7, and neuroticism scores and lower conscientiousness scores. The distribution of participants between Changzhou Dean Hospital and Wuhu Fourth People’s Hospital did not differ significantly according to violent behavior status (χ² = 2.617, P = 0.106). Detailed results are presented in **Table 1**.

**Table 1:** Demographic and clinical characteristics of participants according to violent behavior status (*n* = 202).

Variables	MOAS≥4(n=74)	MOAS<4(n=128)	t/χ^2^	*P*
Age (years)	16.14 ± 1.93	16.10 ± 1.71	-0.179	0.858
Sex			1.903	0.168
Male	22 (29.7)	27 (21.1)		
Female	52 (70.3)	101 (78.9)		
Family history			0.025	0.875
Yes	11 (14.9)	18 (14.1)		
No	63 (85.1)	110 (85.9)		
Educational level			1.304	0.728
Primary school	3 (4.1)	4 (3.1)		
Junior high school	36 (48.6)	53 (41.4)		
Senior high school	31 (41.9)	62 (48.4)		
College	4 (5.4)	9 (7.0)		
First admission			2.478	0.115
Yes	12 (16.2)	33 (25.8)		
No	62 (83.8)	95 (74.2)		
Only-child status			1.294	0.255
Yes	23 (31.1)	50 (39.1)		
No	51 (68.9)	78 (60.9)		
Diagnosis			4.777	0.029
Major depressive disorder	48 (64.9)	101 (78.9)		
Bipolar disorder	26 (35.1)	27 (21.1)		
Single-parent family status			8.994	0.003
Yes	33 (44.6)	31 (24.2)		
No	41 (55.4)	97 (75.8)		
Study center			2.617	0.106
Changzhou Dean Hospital	44 (59.5)	61 (47.7)		
Wuhu Fourth People’s Hospital	30 (40.5)	67 (52.3)		
NSSI score	17.78 ± 10.78	9.94 ± 9.40	5.212	<0.001
GAD-7 score	14.35 ± 6.98	11.62 ± 5.60	2.867	0.005
PHQ-9 score	16.28 ± 7.42	16.42 ± 7.50	-0.127	0.899
Neuroticism	6.94 ± 1.71	6.24 ± 1.39	3.000	0.003
Conscientiousness	4.98 ± 1.63	5.78 ± 1.60	-3.407	0.001
Extraversion	5.62 ± 1.69	5.67 ± 1.62	-0.209	0.835
Openness	7.33 ± 1.79	7.42 ± 1.70	-0.331	0.741
Agreeableness	7.62 ± 1.66	7.47 ± 1.74	0.578	0.564

Data are presented as mean ± standard deviation or n (%). Percentages were calculated within each violent behavior group. Between-group comparisons were performed using the independent-samples t-test for continuous variables and the chi-square test or Fisher’s exact test for categorical variables, as appropriate. MOAS, Modified Overt Aggression Scale; NSSI, non-suicidal self-injury; GAD-7, Generalized Anxiety Disorder-7; PHQ-9, Patient Health Questionnaire-9.

### 3.2 Multivariable logistic regression analysis of factors associated with violent behavior

The primary multivariable logistic regression model simultaneously included study center, age, sex, diagnostic category, single-parent family status, NSSI score, GAD-7 score, neuroticism, and conscientiousness. All nine variables were retained in the model irrespective of their individual statistical significance. No serious multicollinearity was identified among the included variables, with tolerance values ranging from 0.565 to 0.913 and VIF values ranging from 1.095 to 1.770. The omnibus likelihood-ratio test indicated that the overall model was statistically significant (χ² = 67.453, df = 9, P < 0.001). The Hosmer–Lemeshow goodness-of-fit test was not statistically significant (χ² = 10.272, df = 8, P = 0.246), indicating no evidence of poor model fit. Nagelkerke’s R² was 0.388.

After adjustment for the other variables in the model, adolescents from single-parent families had higher odds of violent behavior than those from non-single-parent families (OR = 3.709, 95% CI: 1.535–8.965, P = 0.004). Adolescents with bipolar disorder had higher odds of violent behavior than those with major depressive disorder (OR = 2.879, 95% CI: 1.030–8.046, P = 0.044). Higher NSSI scores (OR = 1.073, 95% CI: 1.033–1.116, P < 0.001) and higher neuroticism scores (OR = 1.322, 95% CI: 1.047–1.670, P = 0.019) were associated with higher odds of violent behavior, whereas higher conscientiousness scores were associated with lower odds of violent behavior (OR = 0.695, 95% CI: 0.552–0.875, P = 0.002).

Male participants had higher odds of violent behavior than female participants (OR = 3.009, 95% CI: 1.321–6.855, P = 0.009). The associations of GAD-7 score, age, and study center with violent behavior were not statistically significant in the adjusted model (all P > 0.05). Detailed results are presented in **Table 2**.

**Table 2:** Multivariable logistic regression analysis of factors associated with violent behavior among hospitalized adolescents with affective disorders.

Variables	B	SE	Waldχ^2^	P	OR	95%CI
Single-parent family status	1.311	0.450	8.478	0.004	3.709	1.535–8.965
Diagnosis	1.058	0.524	4.069	0.044	2.879	1.030–8.046
NSSI	0.071	0.020	12.964	<0.001	1.073	1.033–1.116
Neuroticism	0.279	0.119	5.488	0.019	1.322	1.047–1.670
Conscientiousness	−0.364	0.118	9.546	0.002	0.695	0.552–0.875
GAD-7 score	0.031	0.031	0.989	0.320	1.031	0.970–1.096
Sex	1.102	0.420	6.880	0.009	3.009	1.321–6.855
Age	−0.044	0.136	0.106	0.744	0.957	0.733–1.249
Study center	0.720	0.455	2.507	0.113	2.055	0.843–5.013
Constant	−2.503	2.419	1.071	0.301	0.082	—

The dependent variable was violent behavior, coded as MOAS <4 = 0 and MOAS ≥4 = 1. Single-parent family status was coded as no = 0 (reference) and yes = 1. Diagnosis was coded as major depressive disorder = 1 (reference) and bipolar disorder = 2. Sex was coded as male = 1 and female = 2 (reference). Study center was coded as Changzhou Dean Hospital = 1 and Wuhu Fourth People’s Hospital = 2 (reference). Age, NSSI score, GAD-7 score, neuroticism, and conscientiousness were entered as continuous variables. All nine variables were entered simultaneously using the enter method and retained in the model irrespective of their individual statistical significance. No serious multicollinearity was identified among the included variables (tolerance range: 0.565–0.913; VIF range: 1.095–1.770). NSSI, non-suicidal self-injury; GAD-7, Generalized Anxiety Disorder-7; MOAS, Modified Overt Aggression Scale; OR, odds ratio; CI, confidence interval; VIF, variance inflation factor.

## 4 Discussion

A total of 202 hospitalized adolescents with affective disorders were included in this study, of whom 74 exhibited violent behavior, yielding a prevalence of 36.6%. This rate was notably higher than the 20.2% reported among adolescents in the general population (30). Previous studies have reported that the prevalence of impulsive aggression among hospitalized patients with bipolar disorder is 37.5% (31), while the prevalence of aggressive behavior in psychiatric inpatient settings ranges from 8% to 76% (9). The prevalence observed in the present study is generally consistent with these findings, although direct comparisons should be made cautiously because previous studies have differed in study populations, clinical settings, observation periods, measurement instruments, and definitions of violent or aggressive behavior. In particular, the present study used a MOAS score of ≥4 as an operational cutoff; therefore, the observed prevalence should be interpreted in relation to this definition.

Previous research has identified emotion dysregulation as an important psychological mechanism underlying aggressive behavior in adolescents, with impaired emotion regulation and the accumulation of negative emotions being closely associated with increased aggression (32). Patients with affective disorders often experience difficulties in emotion regulation and impulse control, which may increase their vulnerability to aggressive behavior when confronted with stressful situations. In addition, hospitalized patients typically present with more severe clinical symptoms. In particular, individuals with bipolar disorder may exhibit irritability, heightened impulsivity, and reduced behavioral inhibition during manic or hypomanic episodes, thereby increasing the likelihood of violent behavior (33). Furthermore, adolescence is a developmental period characterized by ongoing psychological maturation. Limited emotion regulation skills and relatively poor social adaptation may make adolescents more likely to respond to stressful events with aggressive behaviors (34). However, emotion regulation, impulsivity, manic symptom severity, and the temporal sequence between these characteristics and violent behavior were not directly examined in the present study. Therefore, these explanations should be regarded as possible interpretations rather than demonstrated mechanisms.

Multivariable logistic regression analysis showed that male sex, single-parent family status, bipolar disorder, higher NSSI scores, and higher neuroticism scores were associated with higher odds of concurrent violent behavior, whereas higher conscientiousness scores were associated with lower odds. These findings indicate that violent behavior among hospitalized adolescents with affective disorders is associated with characteristics across demographic, clinical, behavioral, family-related, and personality-related domains, which is broadly consistent with previous evidence emphasizing the multifactorial nature of aggression in psychiatric inpatient settings (9).However, these associations should not be interpreted as causal effects or as evidence that the identified variables prospectively predict future violent events.

Male participants had higher odds of violent behavior than female participants after multivariable adjustment, although the sex difference was not statistically significant in the unadjusted comparison. Previous reviews of aggression in psychiatric inpatient settings have suggested that sex may be associated with aggressive behavior, although the direction and magnitude of the association have varied across populations and clinical contexts (9, 10). In the present study, the association became apparent after adjustment for diagnostic category, family structure, NSSI, anxiety symptoms, personality traits, age, and study center. This difference between the unadjusted and adjusted results may reflect the influence of covariate adjustment and correlations among the included variables. Because the number of male participants was relatively small, this finding should be interpreted cautiously and confirmed in larger samples.

Anxiety symptoms also warrant consideration. GAD-7 scores were significantly higher among participants with violent behavior in the unadjusted analysis, but the association was no longer statistically significant after adjustment for the other variables. The VIF values for GAD-7 score and neuroticism were 1.390 and 1.114, respectively, indicating that serious multicollinearity was unlikely to account for this result. Instead, anxiety symptoms may share explanatory variance with NSSI, diagnostic category, neuroticism, and other emotional or behavioral characteristics. Specific manifestations such as irritability, agitation, anger, or acute arousal may also be more closely associated with aggression than the general anxiety symptoms measured by the GAD-7. Future studies should assess these symptom dimensions more specifically.

In the present study, higher NSSI scores were independently associated with higher odds of concurrent violent behavior. Previous studies have shown that both NSSI and aggressive behavior are associated with deficits in impulse control and difficulties in emotion regulation, suggesting that they may share common psychopathological mechanisms (35, 36). When individuals are unable to effectively cope with negative emotions, aggression may be directed inward and manifested as self-injury or directed outward and expressed as violent behavior. However, NSSI was assessed over the previous 12 months, whereas violent behavior was assessed cross-sectionally. Therefore, the temporal relationship between NSSI and violent behavior cannot be determined, and NSSI should not be interpreted as a prospective predictor on the basis of the present data. Although the ANSAQ and MOAS assess different constructs and time frames, the MOAS total score includes an autoaggression domain. Therefore, the observed association should be interpreted in relation to the composite MOAS-defined outcome rather than specifically as an association with other-directed violence. Clinically, frequent or severe NSSI may nevertheless indicate the need for a broader assessment of emotion dysregulation, impulsivity, interpersonal conflict, and both self-directed and other-directed harmful behavior.

Regarding personality traits, neuroticism was positively associated with violent behavior, whereas conscientiousness was negatively associated with violent behavior. Individuals with high levels of neuroticism are more likely to experience negative emotions such as anxiety, anger, and hostility and often exhibit poorer emotional stability and impulse control when facing stressful situations, which may partly explain the observed association with violent behavior. Individuals with higher conscientiousness generally demonstrate better self-regulation and stronger adherence to social norms, enabling them to cope with conflicts and frustrations in a more adaptive manner, which may partly explain the observed association with lower odds of violent behavior (37). These findings highlight the importance of considering personality characteristics in a broader clinical assessment of violent behavior. Nevertheless, these explanations remain tentative because each BFI-10 personality dimension was assessed using only two items, and the cross-sectional design does not establish whether these personality characteristics preceded violent behavior.

Family environment was also associated with violent behavior. Adolescents from single-parent families had 3.709 times the odds of concurrent violent behavior compared with those from non-single-parent families. Previous research has shown that family structure and family functioning are closely associated with violent behavior in adolescents. Factors such as single-parent family status, family conflict, ineffective parenting practices, and insufficient emotional support may all be associated with a higher likelihood of aggressive behavior (38). However, single-parent family status itself should not be interpreted as a direct cause of violent behavior. It may coexist with unmeasured circumstances such as economic stress, family conflict, reduced parental supervision, caregiver burden, exposure to family violence, or limited social support. Because these family-level factors were not directly assessed, residual confounding cannot be excluded. The finding should therefore support a more detailed assessment of family functioning and support needs rather than stigmatization based on family structure alone.

With regard to diagnosis, adolescents with bipolar disorder had significantly higher odds of violent behavior than those with major depressive disorder. A systematic review by Aymerich et al. (33) reported that aggressive behavior among children and adolescents with bipolar spectrum disorders is associated with manic symptoms, impulsivity, and emotional instability. Our findings are generally consistent with these observations. During manic or hypomanic episodes, patients with bipolar disorder may present with irritability, heightened impulsivity, and reduced behavioral inhibition, which may increase the likelihood of violent behavior (33). Substance use disorders are also clinically relevant comorbidities in bipolar disorder and may be associated with more complex clinical presentations and treatment responses (39). Because substance use was not systematically assessed in the present study, its potential confounding effect could not be examined. Moreover, the present study did not systematically record current episode type, manic symptom severity, mixed features, acute agitation, or medication exposure. The observed association with diagnostic category may therefore partly reflect these unmeasured clinical characteristics.

The present findings may have practical relevance for comprehensive clinical assessment and multidisciplinary care planning. In adolescent psychiatric inpatient settings, clinicians may consider information concerning sex, diagnostic presentation, NSSI, family circumstances, emotional instability, and self-regulatory personality characteristics when formulating individualized management plans. Such assessment may inform closer observation, emotion-regulation interventions, family assessment, de-escalation planning, and coordination among psychiatrists, nurses, psychologists, and family members. However, these variables should not be used as a standalone scoring system or as a substitute for clinical judgment and structured, dynamic violence-risk assessment. Instruments such as the Dynamic Appraisal of Situational Aggression are designed to assess imminent aggression through repeated evaluation of changing clinical states (19), whereas the present study examined cross-sectional associations with concurrent violent behavior. The two approaches therefore have different purposes, and the present findings should be viewed as complementary contextual information rather than as a validated prediction tool.

This study has several limitations. First, because of its cross-sectional design, causal relationships between the identified factors and violent behavior cannot be established. Longitudinal studies are needed to further clarify these associations. In addition, the variables were assessed in relation to concurrent violent behavior and should not be interpreted as prospective predictors of future violent events. Second, violent behavior was defined using an operational MOAS cutoff of ≥4. Although this cutoff has been used in previous adolescent research, prevalence estimates and group classifications may vary when different instruments, observation periods, or cutoff values are used. Although the MOAS and ANSAQ assess different constructs and time frames, the MOAS total score includes an autoaggression domain. Therefore, limited content overlap related to self-directed harmful behavior cannot be completely excluded, and the observed association between NSSI and violent behavior should be interpreted in relation to the composite MOAS-defined outcome rather than as evidence of an association specifically with other-directed violence. Third, all participants were recruited from two psychiatric inpatient hospitals in China. Although the two-center design provided greater clinical diversity than a single-center study, the findings may not generalize to outpatient populations, community samples, other diagnostic groups, or healthcare systems in other regions. Fourth, only 74 participants met the criterion for violent behavior, and nine predictor parameters were included in the primary multivariable model, corresponding to approximately 8.2 events per parameter. Although no serious multicollinearity or evidence of poor overall model fit was detected, the modest number of events may still limit the precision and stability of the regression coefficients, particularly for estimates with relatively wide confidence intervals. The findings should therefore be interpreted cautiously and replicated in larger independent samples. Fifth, several established correlates of violence were not systematically assessed, including previous violence, substance use, childhood trauma, exposure to family violence, acute agitation, manic symptom severity, and social support. Their omission may have resulted in residual confounding and may partly account for the observed associations with diagnostic category, family structure, NSSI, and personality traits. Sixth, conduct disorder, other disruptive behavior disorders, and emerging personality disorder traits were not systematically evaluated. Therefore, their potential confounding or explanatory roles could not be examined. Persistent depressive disorder was also not separately coded or assessed, and its association with violent behavior could not be determined. Moreover, the cross-sectional design does not support reliable inference regarding temporal mediation, even if these variables had been measured. Finally, NSSI, emotional symptoms, and personality traits were assessed using self-report instruments and may have been affected by recall or reporting bias. The BFI-10 is a brief measure in which each personality dimension is represented by only two items, which may provide less precise assessment than longer personality inventories. Future longitudinal and multicenter studies should incorporate structured diagnostic interviews, detailed histories of violence and trauma, substance-use assessment, family functioning, episode-related clinical variables, and repeated measures of aggressive behavior.

In conclusion, male sex, single-parent family status, bipolar disorder, higher NSSI scores, and higher neuroticism scores were associated with higher odds of concurrent violent behavior among hospitalized adolescents with affective disorders, whereas higher conscientiousness scores were associated with lower odds. These findings highlight the multidimensional demographic, clinical, behavioral, family-related, and personality-related characteristics associated with violent behavior in adolescent psychiatric inpatient settings. They may inform comprehensive clinical assessment and multidisciplinary care planning but should not be interpreted as causal relationships or as evidence of prospective predictive validity. Larger longitudinal and multicenter studies incorporating established violence-related variables are needed to confirm these associations.

## Data Availability

The raw data supporting the conclusions of this article will be made available by the authors, without undue reservation.
